# Adverse events in deceased hospitalised cancer patients as a measure of quality and safety in end-of-life cancer care

**DOI:** 10.1186/s12904-020-00579-0

**Published:** 2020-06-01

**Authors:** Ellinor Christin Haukland, Christian von Plessen, Carsten Nieder, Barthold Vonen

**Affiliations:** 1 Department of Oncology and Palliative Medicine, Nordland Hospital Trust, PO Box 1480, 8092 Bodø, Norway; 2 Institute of Community Medicine, The Arctic University of Norway, PO Box 6, 9038 Tromsø, Norway; 3 Direction Générale de la Santé, Canton Vaud, Switzerland; 4 Unisanté, Direction Générale de la santé, Avenue de Casèrnes 2, 1018 Lausanne, Switzerland; 5 Institute for Clinical Research, University of Southern Denmark, Odense, Denmark; 6 Institute of Clinical Medicine, The Arctic University of Norway, Tromsø, Norway; 7 Centre for Clinical Documentation and Evaluation, Northern Norway Regional Health Authority, PO Box 6, 9038 Tromsø, Norway

**Keywords:** Adverse event, End of life, Palliative care, Global trigger tool, Patient safety

## Abstract

**Background:**

Anticancer treatment exposes patients to negative consequences such as increased toxicity and decreased quality of life, and there are clear guidelines recommending limiting use of aggressive anticancer treatments for patients near end of life. The aim of this study is to investigate the association between anticancer treatment given during the last 30 days of life and adverse events contributing to death and elucidate how adverse events can be used as a measure of quality and safety in end-of-life cancer care.

**Methods:**

Retrospective cohort study of 247 deceased hospitalised cancer patients at three hospitals in Norway in 2012 and 2013. The Global Trigger Tool method were used to identify adverse events. We used Poisson regression and binary logistic regression to compare adverse events and association with use of anticancer treatment given during the last 30 days of life.

**Results:**

30% of deceased hospitalised cancer patients received some kind of anticancer treatment during the last 30 days of life, mainly systemic anticancer treatment. These patients had 62% more adverse events compared to patients not being treated last 30 days, 39 vs. 24 adverse events per 1000 patient days (*p* < 0.001, OR 1.62 (1.23–2.15). They also had twice the odds of an adverse event contributing to death compared to patients without such treatment, 33 vs. 18% (*p* = 0.045, OR 1.85 (1.01–3.36)). Receiving follow up by specialist palliative care reduced the rate of AEs per 1000 patient days in both groups by 29% (*p* = 0.02, IRR 0.71, CI 95% 0.53–0.96).

**Conclusions:**

Anticancer treatment given during the last 30 days of life is associated with a significantly increased rate of adverse events and related mortality. Patients receiving specialist palliative care had significantly fewer adverse events, supporting recommendations of early integration of palliative care in a patient safety perspective.

## Background

Effectiveness and safety are essential elements of value-based cancer care that need to be considered when making decisions about treatment during the entire continuum of the disease [1–3]. Striking the right balance between the two is a major clinical challenge, especially when the disease progresses towards the end of life. At this stage discontinuing anticancer treatment is one of five recommendations to reduce unnecessary treatment and increase the value of healthcare for patients with advanced cancer [4, 5].

Survival is of critical concern for cancer patients, but near the end of life the quality of care and how patients spend their remaining time is just as important [6, 7]. Nevertheless, up to one out of five cancer patients receives anticancer treatment during the last 30 days of life without clear benefit of prolonging survival. The treatment also exposes them to the risk of severe negative consequences such as increased toxicity and decreased quality of life [8–10]. A meta-analysis of the efficacy and safety of anticancer treatment compared to palliative care found no difference in overall survival and significantly more severe adverse events among patients receiving anticancer treatment during the last 30 days of life [11]. This emphasises the need not to focus just on survival, but also the need to assess symptoms, toxicities and complications of anticancer treatment by systematically measuring adverse events [12].

Today, quality measures for end-of-life cancer care generally examine utilisation of healthcare services and use of systemic anticancer treatment, radiotherapy and specialist palliative care during the last month of life [13–15]. Although severe adverse events in cancer care are considered an important outcome measure with high clinical value, current measurements do not include adverse events as an indication of quality and safety in end-of-life cancer care [16].

Thus, the objectives of our study is to investigate the association between anticancer treatment given during the last 30 days of life and adverse events contributing to death and see if adverse events can be used as a measure of quality and safety in end-of-life cancer care.

## Methods

### Study design

The study is a retrospective cohort study of deceased hospitalised cancer patients. We performed a standardised retrospective record review using the Global Trigger Tool (GTT) to identify adverse events contributing to death related to anticancer treatment given during the last 30 days of life.

### Setting

The study was conducted at a public health trust in Northern Norway, providing healthcare to a population of 136,000 inhabitants. Nordland Hospital Trust is a public health trust with three general hospitals; one central teaching hospital and two smaller district hospitals. Cancer patients are treated and hospitalised in all three hospitals, but only the central hospital has a separate oncology and haematology department providing ambulatory chemotherapy and palliative radiotherapy. All three hospitals has a specialist palliative care team providing both inpatient and ambulatory care to patients referred to them. None of the hospitals has a separate oncological inpatient unit. Accordingly, the primary care of hospitalised cancer patients is provided by other specialists (e.g. internist, surgeon and neurologist) depending on the origin of the cancer, who then consults an oncologist or palliative care if needed.

### Study population

The cohort includes all cancer patients with solid tumours and haematological malignancies, 18 years or older who died in one of the three hospitals. Since there were no previous studies indicating incidence rates of adverse events contributing to death in this selected population we did a consecutive sampling of all cancer patients who died in the three hospitals between January 1st 2012 and December 31st 2013. Of the 737 deceased hospitalised patients, 16 children under the age of 18 years were excluded. 247 (34%) patients had cancer as primary or secondary diagnosis on discharge classified by the ICD-10 system. These cancer patients were divided into one group that had received any kind of anticancer treatment and a second group that had not received any anticancer treatment during the last 30 days of life. From the electronic patient records we obtained baseline demographics such as age, gender, length of stay, hospital, department, primary and secondary diagnosis on discharge. We also reviewed the patient records for the type of cancer, presence of metastases, setting (diagnostic, curative or palliative), the last date of administration of parenteral or oral anticancer treatment (chemotherapy, targeted agents and immune therapy), the use of radiotherapy and cancer directed surgery, as well as the date for involvement of specialised palliative care.

### Retrospective review

During 6 months in 2015, a team of two oncology nurses and one oncologist did a structured review of the patient records. The review was conducted according to the Norwegian version of the Institute of Healthcare Improvement GTT manual [17, 18]. The method is a two-stage process where the nurses independently review all records using triggers to identify adverse events. To the 48 general triggers, we added 21 specific oncology triggers developed by the 1000 Lives Plus Campaign in Wales, UK [19]. The two nurses independently identified the presence, category and severity of the AEs, before they discussed their findings with the oncologist and together reached consensus. To validate the results, two other physicians independently re-reviewed the records of adverse events contributing to death and confirmed/rejected the adverse event, severity and type of harm.

### Definition and classification of adverse events

We defined an adverse event as: “Unintended physical injury resulting from or contributed to by medical care that requires additional monitoring, treatment or hospitalization, or that results in death” [18]. The severity of AEs was categorised according to the NCC MERP index [20]. Adverse events were recorded into six main categories: healthcare acquired infections, surgical complications, bleeding/thrombosis, medication harm, pressure ulcer and others. For medication-related adverse events, the generic name was documented.

### Statistical analysis

We summarised the data using descriptive statistics and compared the groups using the Mann-Whitney U test for non-parametric continuous variables, and the Chi square, Fisher’s exact or Linear-by-Linear test for categorical variables. There were no missing data. Incidence rates of adverse events, severities and categories of adverse events were compared using Poisson regression for generalised linear models. Binary logistic regression was used to analyse if adverse events were significantly associated with use of anticancer treatment during the last 30 days of life. Adverse events contributing to death were set as the dependent variable, while use of systemic anticancer treatment during the last 30 days of life was included as a dichotomous explanatory variable. Building a model we first assessed which variables were a potential confounder, before we adjusted for length of stay, age, gender and primary malignancies. To reduce the probability of Type I errors (Bonferronis` correction) the number of variables included were limited to five. A p-value of < 0.05 was considered significant. We used the statistical package IBM SPSS Statistics V.25.0 to analyse the data.

## Results

### Patient characteristics

Most patients had advanced cancer and were in a palliative care setting. Sixty percent of the patients received some kind of anticancer treatment, mainly systemic anticancer treatment. Patients receiving treatment during the last 30 days of life had a longer length of stay and were more often admitted to the central hospital. Patients with lung cancer, lymphoma and haematological malignancies were more likely to receive treatment during the last 30 days of life. Table 1 compare characteristics between patients receiving anticancer treatment during the last 30 days of life with patients not receiving such treatment.

**Table 1:** Characteristics

Variable	No anticancer treatment last 30 days ***n*** = 174		Anticancer treatment given last 30 days ***n*** = 73		***P*** value ^**a**^
	***n***	%	***n***	%	
**Age** (years) - median (min - max)	72	(18–93)	74	(40–91)	NS
**Length of stay** (days) - median (min - max)	8	(0–84)	12	(0–68)	0.03
**Gender**					NS
Female	68	39%	28	38%	
Male	106	61%	45	62%	
**Hospital**					< 0.01
District Hospital Lofoten	27	16%	6	8%	
District Hospital Vesterålen	47	27%	11	15%	
Central Hospital Bodø	100	57%	56	77%	
**Department**					NS
Internal medicine	76	44%	44	60%	
Surgery	91	52%	25	34%	
Others	7	4%	4	6%	
**Primary malignancy**					0.02
Upper gastrointestinal	34	20%	5	7%	
Colorectal	27	15%	4	5%	
Lung	38	22%	21	29%	
Breast	7	4%	4	5%	
Gynaecological	5	3%	2	3%	
Urological	8	5%	6	8%	
Male genitalia	11	6%	4	5%	
Haematological and lymphoma	17	10%	22	30%	
Unknown origin	15	8%	2	3%	
Other ^b^	12	7%	3	4%	
**Treatment intent**					< 0.001
Palliative	135	78%	67	92%	
Curative	2	1%	3	4%	
Diagnostic	37	21%	3	4%	
**Anticancer treatment**					< 0.001
Systemic treatment	64	37%	52	71%	
Radiotherapy	5	3%	14	19%	
Surgery	5	3%	7	10%	
No treatment	100	57%	0	0%	
**Specialist palliative care**					NS
> 30 days before death	33	19%	11	15%	
< 30 days before death	32	18%	14	19%	
Not involved	109	63%	48	66%	

^a^The *p* value measures the difference between the two groups and was set to 0.05. NS not significant

^b^The group consist of patients with cancer from head-neck, sarcoma, malignant melanoma, eye and CNS

### Treatment during the last 30 days of life

Anticancer treatment of any kind was given to 30% of patients during the last 30 days of life. Treatment given during the last 30 days was mainly systemic anticancer treatment (21%). In addition, 8.5% of the patients received radiotherapy during the last 30 days of life, where of more than half during the last 10 days of life. Specialised palliative care was provided equally to both groups, 34 vs. 37% (Table 1).

### Adverse events

**Fig. 1:**
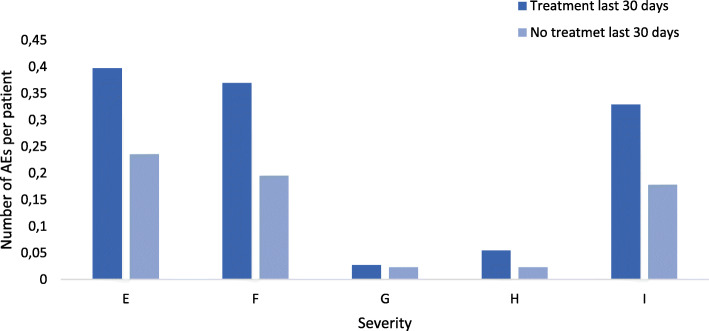
Severity of adverse events per patient categorised according to the NCC MERP Index. Comparing severity of adverse events between patient receiving or not receiving anticancer treatment during the last 30 days of life. Category E: temporary harm that required intervention. Category F: temporary harm that required initial or prolonged hospitalisation. Category G: permanent patient harm. Category H: intervention required necessary to sustain life. Category I: harm contributes to patient death

Patients receiving anticancer treatment during the last 30 days of life had 46% more adverse events than patients not treated during the last 30 days of life, 82 vs. 56 adverse events per 1000 patient days (*p* < 0.01, RR 1.46, CI 95% 1.10–1.94). Patients receiving treatment during the last 30 days of life experienced more temporary adverse events (severity E and F), 25 vs 16 adverse events per 1000 patient days (RR 1.61, *p* = 0.07 CI 95% 1.14–2.27). They also more severe adverse events contributing to death (severity I), 11 vs. 6 adverse events per 1000 patient days, (RR 1.84, *p* = 0.024 CI 95% 1.08–3.14) (Fig. 1). Patients in both groups receiving specialist palliative care had significantly fewer adverse events than patients not referred to palliative care, 52 vs. 73 adverse events per 1000 patient days (RR 0.71, *p* = 0.02 CI 95% 0.53–0.96).

### Types of adverse event

**Fig. 2:**
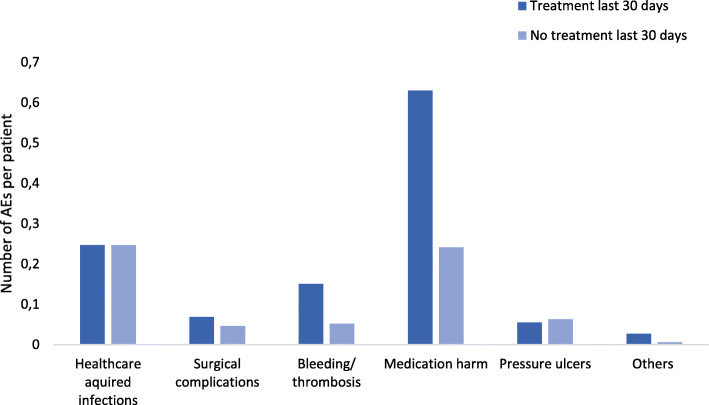
Type of adverse events per patient. Comparing types of adverse events between patient receiving or not receiving anticancer treatment during the last 30 days of life

The most common types of adverse events were healthcare-acquired infections and medication related adverse events (Fig. 2). There was no difference in the rate of healthcare-acquired infections, surgical complications, pressure ulcers or others between the groups. Patients receiving treatment during the last 30 days of life had significantly higher rates of medication related adverse events, 21 vs. 9 adverse events per 1000 patient days (*p* < 0.001, RR 2.35, CI 95% 1.55–3.58). Twenty-four percent of patients receiving systemic anticancer treatment had an adverse event related to the treatment. Bleeding or thrombosis also occurred more often in patients receiving treatment during the last 30 days, 5 vs. 2 adverse events per 1000 patient days (*p* = 0.003, RR 2.62, CI 95% 1.09–6.34). For more detailed description of types of adverse events see supplementary materials.

### Adverse events contributing to death

An adverse event contributed to death in 22% of all deceased hospitalised cancer patients. Patients receiving anticancer treatment during the last 30 days of life experienced nearly double the rate of adverse events contributing to death compared to patients not being treated during the last month of life, 33 vs. 18% (*p* = 0.045, adjusted OR 1.85, CI 95% 1.014–3.359). Table 2 presents unadjusted and adjusted results of the association between anticancer treatment given last 30 days of life and adverse events contributing to death. Adverse events contributing to death were mainly medication harms and healthcare acquired infections. Systemic anticancer treatment contributed to death in 11% of patients receiving systemic anticancer treatment, all given during the last 30 days of life. For patients not receiving treatment during the last 30 days of life, healthcare acquired infections contributed to death for 58% of the patients. An adverse event contributed to death more commonly in patients with lymphoma and haematological malignancies, 27 vs. 13%, (*p* = 0.025, S_res_ 2.1). Radiotherapy did not contribute to the death of any patient.

**Table 2:** Association between anticancer treatment given last 30 days of life and adverse events contributing to death

Variables	AE contributing to death	OR	95% CI	*P* value
**Treatment last 30 days**^a^	**32.9%**	**2.26**	**1.211–4.216**	**0.01**
No treatment last 30 days	17.8%			
**Treatment last 30 days**^b^		**1.85**	**1.014–3.359**	**0.045**
Age		0.97	0.938–0.995	0.021
Length of stay		0.98	0.984–0.962	0.184
Gender		1.84	0.935–3.623	0.047
Type cancer				0.145
Gastrointestinal		0.98	0.370–2.599	0.968
Respiratory		0.68	0.181–2.516	0.559
Breast and gynaecology		0.37	0.130–1.027	0.056
Urinary		0.35	0.132–0.934	0.036
Haematological		0.75	0.244–2.280	0.607
Others				

Binary logistic regression analyses presenting ^a^unadjusted and ^b^adjusted results

## Discussion

There are clear guidelines recommending limiting use of aggressive anticancer treatments for cancer patients near end of life [4, 12]. Still we found that one third of deceased hospitalised cancer patients received some kind of anticancer treatment during the last 30 days of their lives. Patients receiving anticancer treatment during the last 30 days of life also had an increased rate of adverse events compared to cancer patients not given treatment in this period. Most of the adverse events were temporary harms requiring medical intervention, often initiating or prolonging hospitalisation (severity E and F). Even less severe adverse events can cause an extra burden of harm and reduce the quality of life during the limited remaining time, when many patients prefer to be at home with their families [6, 21].

We found that one in five deceased hospitalised cancer patients had an adverse event contributing to death. This included all types of adverse events whether caused by systemic anticancer treatment, other medications or healthcare acquired infections. In a previous study we found that hospitalised cancer patients had an increased risk of adverse events in general compared to other hospitalised patients, and that they more often experienced adverse events related to medications [22].

Our findings are higher than those of registry studies showing that 4–27% of cancer patients die as a complication of anticancer treatment, but these studies do not specifically investigate occurrence of adverse events [14, 15, 23]. We also found that patients receiving anticancer treatment during the last 30 days had twice the odds of having an adverse event contributing to death compared to patients without such treatment. Considering that an adverse event can often be one of many factors contributing to death, it could be that receiving treatment in the last 30 days of life adds yet another layer of treatment related adverse events with an increased risk of hastening death.

Nearly one third of our deceased hospitalised cancer patients received some kind of anticancer treatment during the last 30 days of life, mainly systemic anticancer treatment. Similarly to other studies we found that patients receiving treatment during the last 30 days of life had a longer length of stay, were treated at larger hospitals and more often had lung cancer, lymphoma or haematological malignancies [24–28]. In other studies, the use of anticancer treatment during the last 30 days of life varied from 6 to 43%, depending on country and patients included [29–31]. Our results are consistent with similar studies including all types of malignancies [15, 32], but the rates are higher than in registry studies of solid tumours indicating that Norway has among the lowest (6–10%) use of systemic anticancer treatment during the last 30 days of life in Europe [23, 29]. Thus comparison of the results can be problematic due to differences in study design and included population [13].

Similar to other studies we find that medication harms and healthcare acquired infections were the most common adverse events [22, 33], but their occurrences differed between the groups. While healthcare acquired infections contributed to death of cancer patients in both groups, anticancer treatment related adverse events, contributing to death only occurred in patients who received such treatment during the last 30 days of life. Consequently, when measuring anticancer treatment related adverse events contributing to death we can be more pragmatic and limit the inclusion to deceased hospitalised patients treated during the last 30 days of life.

It is rarely straightforward to argue that anticancer treatment is the direct cause of death. Most likely, reduced functional status, malnutrition and immunosuppression amplify adverse events related to anticancer treatment and increase the negative impact on the patients` remaining lifetime [34]. Our study is not designed to investigate if these treatment-related adverse events affects survival, but nevertheless our results indicate that systemic anticancer treatment given during last 30 days of life can hasten the death of patients.

The proportion of patients treated with radiotherapy during the last 30 days of life in our study, was similar to the results of other studies [23, 35]. While radiotherapy in contrast to systemic anticancer treatment did not contribute to any deaths in our study, it still must be considered of little benefit when given during the last 30 days of life. The benefit of radiotherapy near end of life is questionable with only one out of four patients reporting symptom relief [36]. Patients receiving radiotherapy are also more often hospitalised and die in hospitals [23, 35]. Nearly half of our patients received radiotherapy during the last 10 days of life, which must be considered futile and a misuse of the patients´ time and focus. Radiotherapy can provide needed palliation to patients with advanced cancer, but fractionation regimes should reflect life expectancy and sometimes it is better to provide palliative relief in other ways.

Early referral to palliative care is associated with improved quality of life, fewer acute hospital admissions and less aggressive cancer treatment near the end of life [37–39]. Our findings indicate that patients receiving specialist palliative care had significantly fewer adverse events than patients not referred to palliative care. Symptom management is a key element of palliative care. Diagnosing and managing symptoms at an early stage can prevent them from developing into adverse events and thereby improve the patient safety for cancer patients. This supports recommendations of early integration also in a patient safety perspective. However, our study is not designed to determine if the reduction in adverse events is due to specialised palliative care or due to discontinuing of anticancer treatment.

Even though palliative care should be an integrated part of oncology, patients are often first referred to palliative care when anticancer treatment ends [40]. Knowing the positive associations for the quality of life and safety benefits for cancer patients referred to palliative care, the low referral rate (35%) of deceased cancer patients is worrisome. Availability of specialist palliative care are equal to all cancer types at our hospital and the palliative care teams has regular follow up with all departments. Nevertheless, the culture for referral may vary between specialties. One reason for the low referral to palliative care could be the perception that palliative care is equal to end-of-life care. Since the study was conducted in 2012–2013 this perception has gradually changes and palliative care is increasingly actknowledged as an important part of good quality cancer care that should be integrated early in the course of disease [40].

Other reasons for low referral rates could be resources allocated to palliative care and a healthcare system consisting of silos, not structures to support the integration of palliative care across all specialties and throughout the whole continuum of cancer care. In so means, early referral to palliative care itself can be regarded as a relevant clinical measure of quality in cancer care.

Strength of our study is the completeness of the data. We have included all cancer patients who died during a two-year period at our hospitals. Norway has one of the highest rates of hospital deaths for cancer patients and cancer patients receiving treatment during the last 30 days of their lives are often hospitalised and die in hospital [29, 32]. We therefore argue that our study population is representative of cancer patients cared for by a general hospital trust. But, given the considerable variations in oncology practice within and across countries, the generalizability of our finding can be debated [29]. The main limitation of our study is that it is from only one hospital trust in Norway.

Known limitations of retrospective record reviewing such as information bias and subjective judgments may also apply to our study. Conscious of these limitations we have used a standardised review method (GTT method) with high sensitivity and specificity compared to other methods detecting adverse events [41]. To address limitations with the method of poor to moderate reliability, the review was conducted by a consistent and experienced oncology team [42–44]. In addition, we assessed the validity of our findings by having two physicians independently re-review and verify adverse events contributing to death. We found good correlation between the reviewers, where the severity changed only once and type of adverse event changed twice. However, when studying the intensity and safety of end-of-life care a retrospective design has the advantage since we only know the exact period before death retrospectively. A retrospective design allows for easy identification of cohorts of relevant patients and avoidance of inclusion bias [45].

## Conclusion

Anticancer treatment given during the last 30 days of life is associated with a significantly increased rate of adverse events with twice the odds of having an adverse event contributing to death. Patients receiving specialist palliative care had significantly fewer adverse events, supporting recommendations of early integration of palliative care in a patient safety perspective. Identifying these adverse events is clearly warranted to improve clinical practice and avoid overtreatment in end-of life cancer care. Doing so with a standardised review method on a limited number of deceased hospitalised cancer patients proved to be efficient, and can provide a pragmatic real time measure of quality and safety in end-of-life cancer care.

## Supplementary information

**Additional file 1.**

## Data Availability

The datasets used and analysed during the current study are available from the corresponding author on reasonable request.

## Abbreviations

GTT: Global Trigger Tool
NCC MERP: National Coordinating Council on Medical Error Reporting and Prevention
